# Application of data mining technology in college mental health education

**DOI:** 10.3389/fpsyg.2022.974576

**Published:** 2022-08-11

**Authors:** Xiaocong Sun

**Affiliations:** School of Education Science, Xinxiang University, Xinxiang, China

**Keywords:** data mining technology, college students, mental health education, database technology, decision tree algorithm

## Abstract

In order to improve education and teaching methods and meet the “heart” needs of college students in the era of big data, this paper analyzes the application of data mining technology in college mental health education, and introduces database technology and decision tree algorithm to support college mental health work. This process verifies the feasibility of this kind of system with the help of an example. Using the test standards outlined in this document, 1.5 previous test tasks were completed within the timeframe. During the system test, the error rate was 14% and the number of tests was 7%.However, the error rate in the development stage is 11%, which is lower than 19% of the old version. The error rate in the acceptance stage is 14%, which is lower than 5% of the old version. That is to say, most of the errors were found in time in the system analysis and design stage. 14% of the problems found in the development stage are basically small problems in the interface display, which do not need major changes. However, the old version also includes design defects found in the development stage, and only large-scale rewriting of the involved modules. In the research process, the work of mental health in Colleges and universities has been promoted. At this time, the law of psychological changes of college students has been summarized. Therefore, the support of data mining technology can better meet the needs of mental health education in Colleges and universities.

## Introduction

**FIGURE 1:**
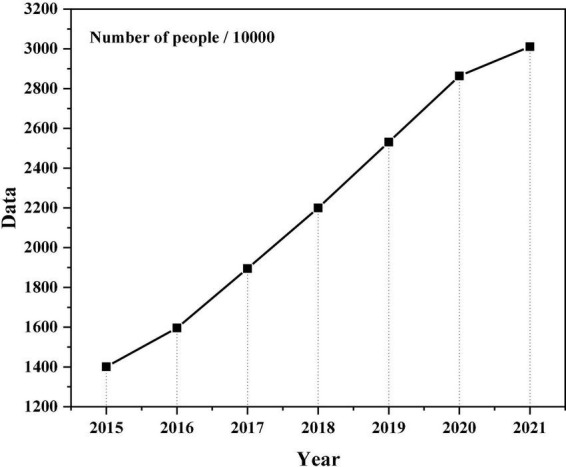
Proportion of college students’ mental health problems.

The rapid growth of information and information technology has brought new ideas and improvements to the traditional mental health education. How to adapt to the development of science and technology in the big data and how to find new ideas and approaches to the health of boys and college girls is a topic that needs to be discussed now. We need to make better use of the internet, rely on big data, use data mining technology as tools, use data platform as a platform, and learn about the “feelings” present of the mind. To drive the “mind” demand for data mining technology, the “mind” type of “big data + psychology education” will be combined to create “mind” platform for the study of mental health combined with the “mind” of the school. The system identifies the “mind” benefits of current activity and seeks to achieve the goal of creating a “mind,” identifying “mind” status of health. Mental illness in the term “mind.” China’s mental health problems are shown in Figure 1 (Jiang et al., 2020).

According to the current situation, a large number of adverse events occur frequently among college students. A series of problems, such as “suicide by jumping off a building,” “Majiajue incident,” “online love” and “Internet addiction,” which are frequently reported in the newspapers, have sounded the alarm for people—the top students in the ivory tower are ill and seriously ill (Zheng and Zhou, 2021). The health of college students is a concern throughout the community, because college students are the last stage of national education planning, and they performance determines the level of technological development in our country. They are not only about the future of the country, but also about the development of the country. Their expertise determines the speed and quality of China’s reform process, and its overall impact on China’s future. Only by examining the current state of mental health of college students in China today in a timely and legal manner can we solve their mental health problems. According to the goals and objectives and the development of new technologies in our country (Sang, 2021).

Based on the above criteria, this article has been selected as a tool to assess the mental health of two new students from various regions to better understand mental illness of modern college students in China. It also helps them deal with their mental health problems in a timely manner, take care of their health and wellbeing, have a positive outlook on life, complete their education, complete their education and work for their country. At the same time, we hope that the results of this study will be of great benefit to the relevant agencies of China in developing measures affecting the mental health of young men. The study focused on specific areas and had statistical data of 4738. 63 percent of boys are 2,983 and 1,755 or 37 percent of girls. Highlights of student resources: major cities are the top cities in the state, small cities and middle are state-level cities and towns, and cities are cities, cities, and towns. Of these, 1,174 came from major cities, accounting for 24.8% of the total students, 1,575 students from small and medium-sized cities, 33.2%, and 1,989 students from the population, rural, 42% (see Table 1; Meng et al., 2020).

**TABLE 1:** Statistics of surveyed students.

	Boys	Girls	Big city	Small and medium-sized city	Countryside
Number (person)	2,983	1,755	1,174	1,575	1,989
Percentage%	63	37	24.8	33.2	42

Symptom checklist was used in the investigation. The scale contains 9 factors and 90 items, including physical maladjustment, interpersonal relationship, depression, anxiety, hostility, terror and so on. The validity of the questionnaire was *v* = 0.78 and the reliability was a = 0.83. Learn about the state of mental health in modern colleges. Published 4,752 questionnaires and scored 4,738 points. The return rate is 99.7%, valid and 100%. Conduct an analysis of current data on impact issues, document, compare and compile data on current issues, and create scenarios for further research (Barrett and Twycross, 2020; Wan and Wang, 2021).

## Literature review

**FIGURE 2:**
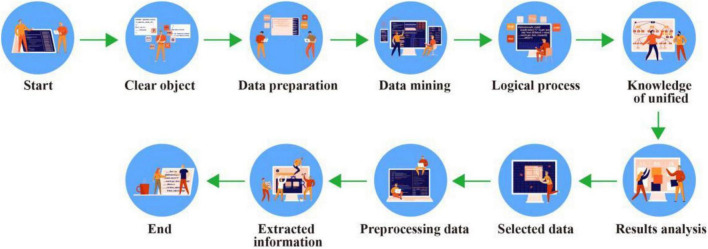
Data mining technology process.

Data mining is often associated with computer science, and these goals can be achieved in a number of ways, including statistics, data recovery, validation standards, analysis. Online, professional, and technology training. Many problems have arisen since the development of data mining technology. The following describes several different definitions of data mining: One definition is the extraction of a hidden, previously unknown, important piece of data; another topic is research to extract useful information from multiple sources or data. The goal of data mining is to effectively organize the existing information and obtain valuable and difficult to find information and the association between information. It is based on many disciplines such as statistics and artificial intelligence. It realizes intelligent analysis of data, infers and summarizes information, finds potential laws, predicts customer behavior, helps the organization’s decision makers correctly predict upcoming scenarios, adjust strategies, reduce risks, and make correct decisions (Zhou and Yang, 2021). Data mining is often used in data analysis, such as intelligence, but it is a rich term used in many ways. Its relationships to KDD are: KDD is the process of identifying models that are cost-effective, innovative, cost-effective, and ultimately understandable from data (Liu et al., 2021); Data mining means that KDD generates special data in the agreement including constraints by a special algorithm. In fact, these two terms are often used illegally in modern literature. The structure of data mining technology is shown in Figure 2 (Li, 2021; Zhang, 2021).

**FIGURE 3:**
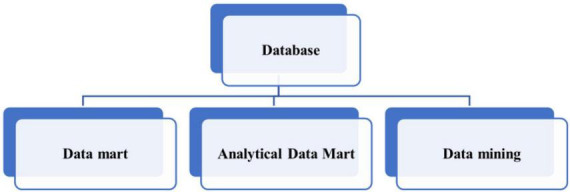
Data mining library obtained from data warehouse.

Previously unpredicted information refers to the information that has not been and is not easy to speculate or guess. That is, one of the tasks of data mining is to find the internal relations that are not easy to rely on empirical reasoning, or even the experience or knowledge that is contrary to intuition. However, once the information mined is more contrary to people’s experience, it is more likely to be more valuable. The most classic example of the application of data mining in real life is that a chain store found the close relationship between children’s diapers and beer in data mining. Data mining and data warehouse have a very important relationship. In most cases, data mining must first import data from the data warehouse into the data mining library or data mart, as shown in Figure 3 (Bai and Zhou, 2021; Ngussa et al., 2021). For example, the inconsistency of all data will not occur. Therefore, the organic combination of data warehouse and data mining will greatly improve the ability of enterprises and institutions to reorganize and reuse information, so that information can better serve decision-making.

Unsupervised learning, description and clustering, supervised learning, shopping basket analysis and relationship grouping are all important methods of data mining. Among them, supervised learning method includes classification, prediction and estimation. Data mining often uses web page mining, deviation analysis, association rules, features, changes, clustering and other methods in data analysis. These methods can enable data mining to mine data from different aspects. These data mining methods are introduced below. *Classification method*. Classification is to compare the similarity of data objects in the database and classify the data with the same or similar characteristics into a set. In this way, the data in the database can be mapped to different sets through the classification method. *Regression analysis method*. Regression analysis is an analytical method to clarify the relationship between multiple variables. Cluster analysis method. The first step of cluster analysis is to divide a group of data into several different categories. *Association rule method*. Association rule method is usually used to describe the degree of association between various data items in the database, that is, to judge the probability that the occurrence of an item in a transaction may cause the occurrence of other items in the transaction, that is, to mine the internal association between data. Feature analysis method. The function of feature analysis method is to extract the features of the data in the database and express them in a suitable way. *Change and deviation analysis method*. There are a lot of potentially interesting knowledge involved in the change and bias analysis method. Its purpose is to find the difference between the observed results and the reference quantity (Iammarino et al., 2020).

**FIGURE 4:**
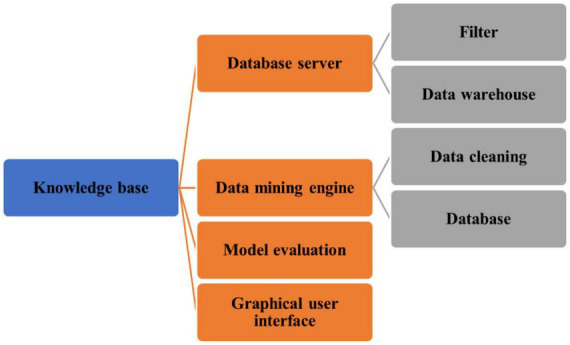
Data mining system structure.

Data mining, also known as information mining, uses automatic or semi-automatic methods to find potential and valuable information and rules in data. Data mining technology comes from database, statistics and artificial intelligence. The composition of data mining system mainly includes the following aspects: Data mining is an important step in the process of learning about data. Knowledge acquisition consists of the following steps: Data cleaning, data collection, data selection, data transfer, data mining, data analysis, and instruction. A similar model for data is only shown in Figure 4 (Aronson and Jaffal, 2022).

## Materials and methods

### Solutions

#### Overall system architecture

**FIGURE 5:**
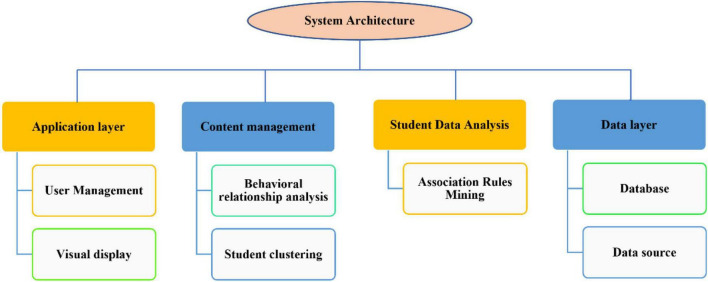
System structure.

Based on the in-depth study of data mining technologies such as cluster analysis and association rule mining, and based on the demand analysis of student behavior, a student behavior analysis system based on cluster analysis. We can have a comprehensive understanding of students, provide decision support for students, and have self-evident practical significance for further understanding students’ behavior characteristics and decision support of school management. For the convenience of function display and user interaction, the system adopts b\s architecture, and the system structure is shown in Figure 5 (Soares et al., 2022). It mainly includes data layer, processing layer and application layer. The specific features are as follows: the data comes from student scores, consumption records and personal information, providing a data basis for subsequent analysis. The processing layer is the core business part of the system, which is responsible for processing the requests of the browser and realizing the data analysis functions such as clustering analysis of student data and association rule mining. Realize user registration, login and display of data analysis results.

The overall framework of the system starts with each data source in the school data, and stores the data in each data source in our database after preprocessing and integration. In the processing layer, the data in the database is extracted, different behavior data are selected through the behavior division module, and the cluster analysis technology is used to cluster the students from different behavior angles, so as to obtain the classification of all students in different behaviors, and have a clear division of the student group. Then, in the behavior association analysis module, combined with the clustering results and the original student data, combined the student behavior characteristics, and used the association rule mining technology to analyze, extract the frequent patterns between different behaviors, and find the association relationship between different behaviors. Then, the results of the student division module and the behavior association analysis module are displayed in the application layer through visualization technology for users to observe and analyze (Lindell et al., 2021; Berman et al., 2022).

#### Database design

Database is a very important part of the system. It is responsible for the storage and management of the data required by the system. Only with the support of database can the corresponding functions of the system be completed. The system processes the data through corresponding scripts. First, clean the data in the data source to remove the null data in the data source. For more effective data analysis, the system converts some data in the data source. For example, the student gender “male” and “female” are replaced by “1” and “0,” respectively. Since the student grades in the data source record the grades of each course respectively, we average the students’ grades in each semester, and then record them in the database. At this time, we also integrate the student consumption data. According to the architecture of the student behavior analysis system, in order to complete the corresponding functions, the database is designed, with a total of 8 tables. The following describes the tables and their relationships. The field description of the student basic information table is shown in Table 2 (Posselt, 2021).

**TABLE 2:** Field description of student basic information table.

Field name	Field type	Field description
SID	VARCHAR(255)	Student ID, primary key
Sex	Int	Gender, 1 for male, 0 for female
College	Int(255)	College to which the student belongs
Start-year	Timestamp	Year of enrollment

### Experimental method of validation scheme

#### Decision tree algorithm

Data mining processes include structured thinking, decision trees, genetic algorithms, neural networks, and more. There are three types of wood cutting. The algorithm is based on data theory and uses levels of data entropy and data gain as a standard measure for using inductive data distribution. The training set S (Kondo et al., 2021).


(1) (S)=∑-p(I)2p(I))

Here P (I) is the proportion of S belonging to class I. Σ is the sum of C. If all s belong to the same class, the range is 0 (classification completed) to 1 (completely random). Note: S is not only an attribute but also the entire sample set.


(2) $$\left(S,A\right)=\sum \frac{\left|S_{v}\right|}{\left|S\right|}\times \left(S_{v}\right)$$

Here Σ is all possible values V of attribute A; Sv = subset of S with v value for attribute A, —Sv— = number of elements in SV,—S— = number of elements in S.

(S, A) is the information gain of attribute A on subset S, which is defined as


(3) (S,A)=(S)-(S,A)

(T, A) refers to the reduction of entropy after the value of attribute a is known. The larger the Gain (S, A), the more information the test attribute a provides for classification (Cage and Howes, 2020).

#### Data mining

During data mining, it is first necessary to create an appropriate determination tree using the algorithm, and first determine the number of positive P samples and the number of negative structure n. The top 15 students with good grades are defined as positive examples, and the last 35 students with poor grades are defined as negative examples. Combined with the above settings, the following formula can be obtained:


(4) P=15,N=35

(5) $$I\,\left(p,n\right)=-\frac{15}{50_{2}}\,\frac{15}{150}-\frac{35}{50_{2}}\,\frac{35}{50}=0.881$$

(6) $$E=\frac{24}{50}\,I\,\left(p^{1},n\right)+\frac{7}{50}\,I\,\left(p^{2},n^{2}\right)+\frac{19}{50}\,I\,\left(p^{3},n^{3}\right)=\frac{24}{50}\,I\,\left(11,13\right)+\frac{7}{50}\,I\,\left(4,3\right)+\frac{19}{50}\,I\,\left(0,19\right)=0.616$$

*I*(*p*, *n*) = −*E* = 0.881−0.616 = 0.265(7)Extra points for experiment report

*I*(*p*, *n*) = −*E* = 0.881−0.801 = 0.08(8)Extra points for experiment class

*I*(*p*, *n*) = −*E* = 0.881−0.879 = 0.002(9)Extra points for final assessment

**FIGURE 6:**
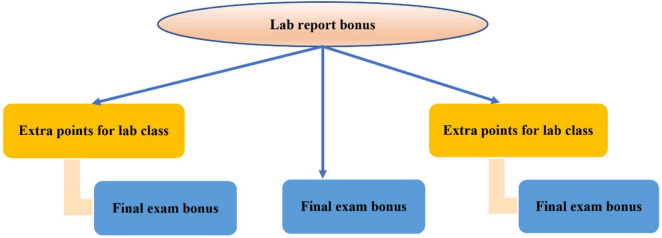
Score analysis decision tree.

According to the calculation, the experimental report bonus has the maximum information gain, so the experimental report bonus is selected as the root node and expanded downward to finally generate the decision tree, as shown in Figure 6 (Xu et al., 2021).

#### Clustering algorithm

In general, the important method used by people to understand things is to classify the objects of knowledge. Things divided into the same class tend to have more similar characteristics. The so-called clustering is not a descriptive task to divide a group of objects into a series of meaningful subsets according to the similarity of objects without training data samples (Bradha et al., 2021). The classical partition clustering methods include k-means algorithm, PAM algorithm, Clara algorithm and clarans algorithm. The classical clustering algorithm is K-means and extended algorithm, which divides object d into a group of clusters:


(7) {C,1C…2C}k

Here:


(8) $$\overset{k}{\underset{i=1}{U}}t\,i=D$$

Where K is the number of clusters to be obtained. This algorithm only classifies an object into one cluster at most, and each cluster is a subset of all objects.

**FIGURE 7:**
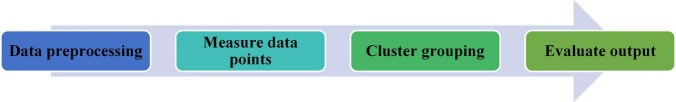
Clustering analysis algorithm.

Using cluster analysis algorithm to analyze students’ archives can provide a strong scientific basis for the management of college students. It can also adopt the way of questionnaire to students, and cluster analysis and processing the students’ personal preferences, living habits, learning habits and other information, so as to provide scientific basis for school management and facilitate scientific management. The main steps of clustering analysis algorithm are shown in Figure 7 (Liu et al., 2015).

#### Test case

**FIGURE 8:**
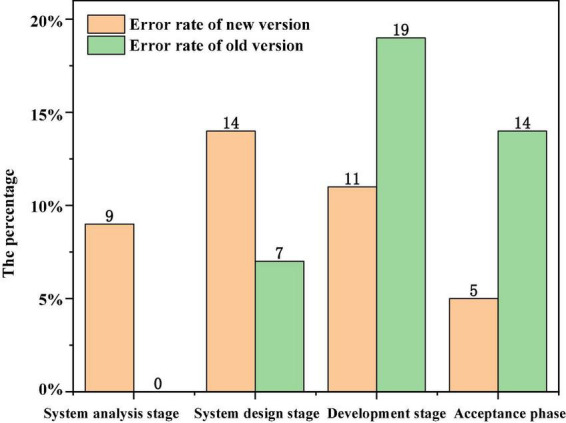
Data comparison between old and new software in different software development periods.

During the development of a new version of an information management platform, the improved model mentioned in this paper is used. According to experience, the development cycle of the new version takes 1 month, half of which is for various tests. In the test of the project, the problems faced are that the new functions to be added are twice as many as those of the previous version, and several modules need to design more in-depth test cases to rewrite the original function modules on a large scale. Neither the personnel nor time invested in testing can increase the time required by users (Huang et al., 2021). The software test engineer stepped in, conducted demand-based tests on the business requirements put forward by customers, and developed acceptance standards with customers at the same time. During system design, software test engineers and system designers work together to write technical test cases and acceptance based test cases, find out defects and errors in the design, and avoid bringing errors in the design into the software development stage. Because such errors are often fatal and cannot be remedied, unit tests and related integration tests will be carried out once the program fragments are written in the development phase, and software changes are tested and developed repeatedly and alternately. However, exploratory testing has found some unpredictable errors to some extent. Special attention is paid to that in the whole testing process, technical testing and acceptance testing are always independent routes due to their different purposes, which ensures the quality of the software. Compared with the old version, the whole software development period is shortened, the software development cost is reduced, the customer’s satisfaction with the software is greatly improved, and the later maintenance work of the software is greatly reduced, which frees the company from a large number of later maintenance work. Figure 8 data comparison between old and new software in different software development periods (Xie et al., 2021).

The error rate in the system analysis stage is 14%, and the number of test cases is 7%. However, the error rate in the development stage is 11%, which is lower than 19% of the old version. The error rate in the acceptance stage is 14%, which is lower than 5% of the old version. That is to say, most errors have been found in time in the system analysis and design stage. 14% of the problems found in the development stage are basically small problems in the interface display, which do not need major changes. However, the old version also includes design defects found in the development stage, and only large-scale rewriting of the involved modules (see Table 3 for details).

**TABLE 3:** Specific data.

	System analysis phase	System design stage	Development phase	Acceptance phase
Error rate of new version	9%	14%	11%	5%
Error rate of old version		7%	19%	14%

## Results

To address the shortage of mental health educators, strengthen the capacity of educators, improve the quality and effectiveness of staff, and improve the quality of education. standard current operation; Changing existing teaching strategies, improving instruction, providing instructional materials and instruction, changing international assignments, increasing grades, completing classes in college, and increase teaching hours. Activities for practicing student skills; Change the form of teachers’ classes and keep the course content up to date. Teachers can adopt the online + offline course learning mode, and the offline courses can independently choose the content of mental health education; On the other hand, it can break the time and space constraints, establish an online communication and teaching platform, timely understand students’ psychological status, and provide psychological related services online, so as to achieve point-to-point, heart-to-heart and practical psychological early warning and consultation, enhance the “heart” effectiveness of psychological education, and make the results of psychological education work benefit more teachers and students.

**FIGURE 9:**
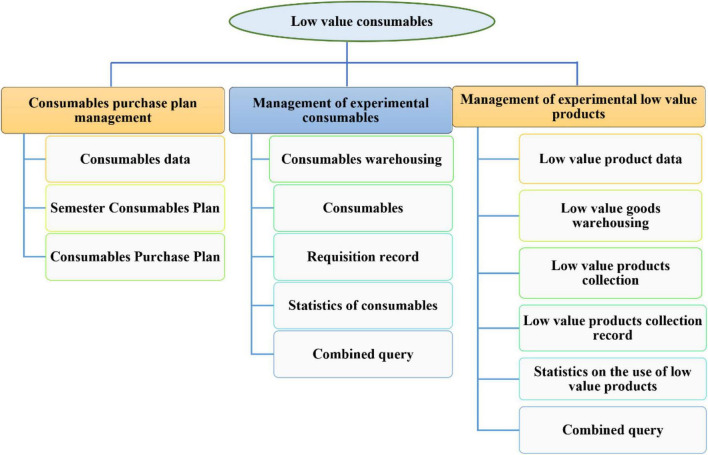
Teaching management system (laboratory part).

Integrating data resources is to effectively combine students’ basic family information, learning of teaching management system, campus card consumption, campus network browsing, campus life track and other relevant data. In the first stage, we collect basic information, and in the second stage, we analyze important data to assess, identify, and classify the characteristics of mental illness and the patterns of students. College, developing mental health information for college students, and collaborating. Creating and sharing mental health information. The data obtained through the screening and analysis platform has expanded the screening indicators of students’ mental health education screening, and promoted the dynamic supervision of students’ psychological early warning data; On the other hand, an early warning and tracking mechanism can be established. Teachers can use the relationship between relevant data to visually observe the data comparison of students under normal and abnormal conditions by integrating various data resources. On this basis, through the comparison and analysis of data, we can formulate relevant counseling and intervention programs for students who may have psychological problems, timely help students adjust themselves, effectively improve the identification of psychological crisis and the efficiency and quality of mental health education, provide personalized services for mental health education, and highlight the “heart” characteristics of mental health education. The teaching management system (laboratory part) is shown in Figure 9.

**FIGURE 10:**
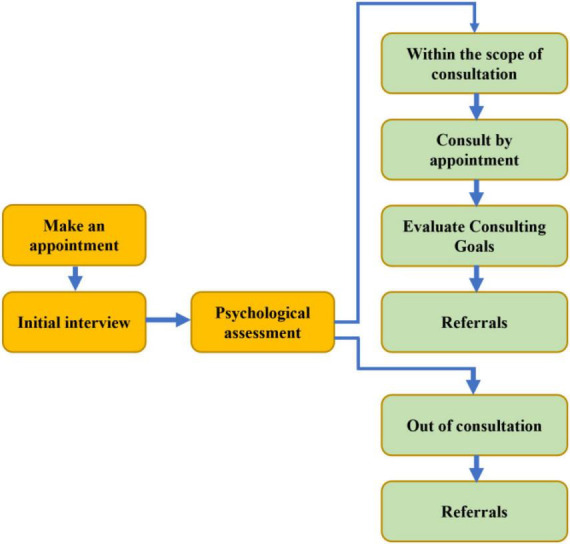
Psychological counseling and counseling process.

At present, the “Post-95” and “Post-00” students in Colleges and universities are in the majority. They are more inclined to obtain information, understand information and remove doubts from the Internet, so they will turn to the Internet at the first time. The creation of a new media platform is in line with the “heart” demand of the rapid development of new media at present. It also injects the “heart” concept and “heart” vitality into the mental health education in Colleges and universities, and creates a “heart” platform for students’ psychological help and consultation at the first time. We will develop a “heart” system for mental health education, and implement the management of four levels of grid mental health supervision and screening: schools, colleges, classes and dormitories. The head of each dormitory is responsible for filling in the daily psychological barometer of the dormitory members. The psychological committee members of each class are responsible for collecting and checking the psychological conditions of students in each class every week. The college plans and manages the psychological conditions of students in the college. The school (psychological center) is responsible for psychological counseling and counseling of extremely abnormal students (Figure 10). Finally, the daily, weekly and monthly reports are formed to open up the last kilometer of mental health education in the school. Through the psychological survey results and relevant resources of students in the University, integrate the data for reasonable and scientific analysis. Push more targeted learning, life, interpersonal psychology knowledge, self-emotion regulation and stress relief adjustment methods through the new media platform.

Through the big data analysis of knowledge learning and students’ mental health on the new media platform for mental health education, some psychological activities can be planned regularly, such as student psychological activity reception day, counselor psychological salon, sand table group counseling, psychological quality development activities, psychological drama situational drama competition, 21 day mental health punch in activities, so as to achieve the effect of new media publicity. Through these activities, it is helpful to build a bridge between teachers and students and improve the effect of mental health practical education.

## Conclusion

Colleges and universities need to improve their knowledge of mental health, improve their education and teaching, move from the “mental” mode to “big data + knowledge” in real time, and create a “spiritual” bridge for students. Meets the “critical thinking” needs of college students when it comes to mental health and education. Psychiatrists use this information to inform psychiatrists about their health, to compensate for the lack of mental illness during the “high” in colleges and universities, serve as the spiritual protector of the growth of students, and protect the “high” life. The spirit and “mind” of high school students creates a “trust” environment that puts their energy and education to work in college. After more than 10 years of development and refinement, data mining technology has been successfully developed in many fields. In today’s world of science, technology, and more and more, it is very difficult to find data that can be used to determine big data-based research. *Research, as always with standard procedures*. Encourage research and decision-making knowledge, based on data mining technology to use data mining technology to extract knowledge and data from multiple data and real laws really hidden inside information.

## Data availability statement

The original contributions presented in this study are included in the article/supplementary material, further inquiries can be directed to the corresponding author.

## Author contributions

XS contributed to the writing of the manuscript and data analysis, supervised the work, and designed the study.
